# Is There Any Advantage of Treating Partners in *Helicobacter pylori* Eradication?

**DOI:** 10.1155/2015/706507

**Published:** 2015-03-15

**Authors:** Halil Rakici, Remzi Adnan Akdogan, Teslime Ayaz, Recep Bedir

**Affiliations:** ^1^Department of Gastroenterology, School of Medicine, Recep Tayyip Erdogan University, 53 100 Rize, Turkey; ^2^Department of Internal Medicine, School of Medicine, Recep Tayyip Erdogan University, 53 100 Rize, Turkey; ^3^Department of Pathology, School of Medicine, Recep Tayyip Erdogan University, 53 100 Rize, Turkey

## Abstract

*Aim.* We designed this trial to find answers to the following questions. (1) Does the success rate decrease in a country where HP prevalence is high? (2) Can we provide benefit by simultaneously treating the partners of infected patients?* Materials and Methods.* The first group consisted of 102 HP-positive patients, and both the patients and their HP-positive partners were treated. The second group consisted of 104 HP-positive patients whose partners were HP-positive but only the patients were treated. The participants in both groups were treated with levofloxacin 500 mg daily, amoxicillin 1 g b.i.d, and lansoprazole 30 mg b.i.d (LAL) for ten days.* Results.* In the per-protocol analysis, the eradication success rate was found to be 92.2% (94/8) in the first group and 90.4% (94/10) in the second group. No statistically significant difference was found between the two groups (*P* > 0.05).* Conclusions.* With regard to the HP eradication rate, no difference was found between treating the HP-positive partners of HP-positive patients simultaneously and not treating them simultaneously. According to these results, we can say that reinfections between partners do not significantly contribute to the failure of eradication.

## 1. Introduction

*Helicobacter pylori* (HP) infection is one of the most prevalent chronic infections in humans. Approximately 50% of the world's population is infected with this bacterium. HP may be an important public health problem because it plays a role in gastritis, peptic ulcer, gastric cancer, and mucosa-associated lymphoid tissue (MALT) lymphoma etiology. The bacterium is usually caught in childhood, and it may follow a course of spontaneous elimination and reinfection. In developing countries, its prevalence is up to 80% in individuals over the age of 50. While reinfections occur more frequently in childhood, infections or reinfections occur less frequently in adulthood.

During childhood, the risk of getting infected is associated with familial living conditions and socioeconomic status. Crowded households, a large number of children, children sharing the same bed, and lack of hot-water or stream water increase the risk of infection. The prevalence decreases as the quality of life increases. For instance, while the prevalence in Japan is 70% in people born before 1950, it is 25% in people born after 1970. Humans seem to be the largest reservoir for HP.

Human-to-human contagion occurs via oral-oral, fecal-oral, and gastro-oral routes. Contagion may occur between partners, between siblings, and from mother to child. Infected gastric materials are a risk factor for contagion. Users of infected gastric materials, endoscopic materials, and other tools can also be infected. Swimming in contaminated water or using (for drinking, cooking, bathing, and washing clothing) that water may also cause infection [1–6]. While reinfections are rare in adults, we designed this study to answer the question as to whether reinfections cause failure in eradication treatment because of contagion between partners.

## 2. Materials and Methods

A total of 664 patients who presented to the Recep Tayyip Erdoğan (RTE) University Gastroenterology Polyclinic between January 2013 and May 2014 were screened for* Helicobacter pylori*. The patients were randomized into two groups, prospectively. There were 308 patients treated in 2 groups. In the first group there were 102 patients and 102 partners of these patients. In the second group there were 104 patients. Ethics Committee Approval from RTE University was obtained. The partners of those HP-positive patients, whose upper gastrointestinal system (GIS) endoscopies revealed that they were also HP-positive, were invited to the clinic, and antigen stool tests were performed on them. A total of 308 patients who were HP-positive and who also had HP-positive partners were included in the study. The participants were divided into two groups. In the first group, 102 HP-positive patients and their HP-positive partners received treatment. In the second group, 104 HP-positive patients were treated, but their HP-positive partners were not treated. Endoscopic biopsies were performed in the Pathology Laboratory of RTE University, and antigen stool tests were performed in a microbiology laboratory at the same university using a Rapid Strip HPSA Kit (Meridian Bioscience Europa, Milan, Italy). Both groups were treated with levofloxacin 500 mg daily, amoxicillin 1 g b.i.d, and lansoprazole 30 mg b.i.d (LAL) for ten days. HP was investigated with an antigen stool test six weeks after treatment. Both groups were investigated to determine any difference in HP eradication rates.

Statistical analyses were performed using power analysis and SPSS software, version 11.5. Intergroup comparisons were made by using Pearson's chi-square test and the Mann-Whitney *U* test. The statistical evaluation included patients who came for a control visit per protocol (PP) and all patients who were randomized (intent-to-treat (ITT)). The power analysis of the research was made using the Minitab 13.0 program and minimum sample size was calculated as *n*: 50 (*α*: 0.05, power: 0.80).

## 3. Results

In the first group, 56 of the 102 patients who completed the study were female and 46 were male. Their age average was 47.5 (20–75). In this group, of the patients whose partners received treatment, 46 were female and 56 were male. Two patients did not complete the study. In the first group, the patients' age average whose partners received treatment was 47 (20–74). In the second group, 58 of the 104 patients who completed the study were female and 46 were male. Their age average was 48.5 (21–76). Two patients did not complete the study. There was no statistically significant difference between the two groups with regard to age and sex (Table 1). In the first group, when* Helicobacter pylori* was investigated with an antigen stool test six weeks after the treatment, it was found to be positive in 94 patients and negative in 8 patients (92.2%). In the treated partners of this group, 92 of the 102 patients were found to be HP-negative, and ten of them were found to be HP-positive (90.2%). In the second group (the control group whose partners were not treated), 94 of the 104 patients were found to be HP-negative and ten of them were found to be HP-positive (90.4%). There was no statistically significant difference with regard to eradication rate between the two groups (*P* > 0.05). The findings are shown in Table 2.

## 4. Discussion

Various ways are sought to increase treatment success in* Helicobacter pylori* eradication. According to the Maastricht IV consensus report, while standard and consecutive treatments are suggested as first-line treatment for* Helicobacter pylori* eradication, standard treatment should not be given in places where clarithromycin resistance is higher than 20%. Different antibiotics (clarithromycin, metronidazole, amoxicillin, tetracycline, furazolidone, rifaximin, levofloxacin, moxifloxacin, sitafloxacin, tinidazole, and rifabutin) and bismuth salts have been used in various combinations and treatment protocols for* Helicobacter pylori* eradication, together with proton pump inhibitors (PPIs). Furthermore, probiotics and antioxidant drugs have been used to increase the success rate of treatment. Actions for new treatment protocols and various factors related to the type of bacteria and the host are also investigated. In spite of this, the desired rate of eradication success has still not been achieved because of antibiotic resistance, toxicity, and compliance problems [7–10]. HP infection is the most common bacterial disease in the world. The important preparatory factor related to HP infection in adulthood is being for lower socioeconomic classes; in childhood those factors include poor living conditions, crowded families, and crowded social settings. Dental plaque in humans and in animals, like pigs and cats, and water infected with feces are sources for the disease. Contagion can occur via fecal-oral, gastro-oral, and oral-oral routes. Therefore, intrafamilial transmission and contagion are important with regard to eradication of this bacterial infection. Reinfection and recrudescence that develop after effective treatment can permanently prevent the ability to eradicate the infection. Low antibiotic efficiency provides temporary clearance rather than eradication. Although it is known that the reinfection rate is low (3.5%) in developed countries, this rate is higher (7.5%) in developing countries and in countries with high HP prevalence [11–16]. HP reinfection usually occurs in the first months after treatment and noticeably reduces one year after treatment. While some authors have suggested that partners are not reservoirs for reinfection, other authors have suggested that this possibility cannot be definitely discarded [17–20].

In this study, we aimed to first determine whether there is a difference between eradication rates regarding treatments with and without partners given to patients with HP whose partners were also infected. We also aimed to compare reinfection and recrudescence rates in long-term follow-ups. We chose the LAL treatment protocol that has been shown to be highly effective (82–91%) as the* Helicobacter pylori* eradication treatment [21–24]. Therefore, 664 patients were screened to reach a sufficient number of HP-positive patients with HP-positive partners. The percentage (31%) of the HP-positive partners of the patients found to be HP-positive, who were invited for treatment with their partners, was significantly lower than the country-wide* Helicobacter pylori* incidence in Turkey. It was reported that the reinfection rate was low at the beginning of the 2000s in Turkey [25]. As a result, no difference was found in the eradication rates of the HP-positive patients treated alone and the HP-positive patients treated with their partners. Based on these results, we can conclude that there is no need to treat infected partners of infected patients, according to the Maastricht IV criteria. In other words, treatment of infected partners does not increase the HP eradication success rate.

Consequently, we can say that calling partners of HP-positive patients, testing them for HP, and treating them, if they are found to be HP-positive, are not a practical or efficient approach. Determining whether there is any difference between patients and controls groups with regard to reinfection and recrudescence in long-term follow-ups is the next objective of the study. The decision to administer HP eradication treatment should be made for each patient, individually, according to the guidelines.

## Figures and Tables

**Table 1:** Basic characteristics of the patients.

Characteristics	Groups	
	Group 1	Group 2
Age	27–68 (44.41)	21–70 (46.21)
Sex		
Female	60 (58.8)	58 (55.8)
Male	42 (42.2)	46 (44.2)

**Table 2:** Eradication rates of the groups.

Groups	Eradication rate			
	ITT analysis		PP analysis	*P* value
Group 1	HP (+)	10 (9.6%)	8 (7.8%)	*P* > 0.05
	HP (−)	94 (90.4%)	94 (92.2%)	

Partners of Group 1	HP (+)	10 (9.6%)	10 (9.8%)	
	HP (−)	94 (90.4%)	92 (90.2%)	

Group 2	HP (+)	12 (11.3%)	10 (9.6%)	*P* > 0.05
	HP (−)	94 (88.7)	94 (90.4)	
